# The Proper Splicing of RNAi Factors Is Critical for Pericentric Heterochromatin Assembly in Fission Yeast

**DOI:** 10.1371/journal.pgen.1004334

**Published:** 2014-05-29

**Authors:** Scott P. Kallgren, Stuart Andrews, Xavier Tadeo, Haitong Hou, James J. Moresco, Patricia G. Tu, John R. Yates, Peter L. Nagy, Songtao Jia

**Affiliations:** 1 Department of Biological Sciences, Columbia University, New York, New York, United States of America; 2 Department of Pathology and Cell Biology, Columbia University College of Physicians and Surgeons, New York, New York, United States of America; 3 Department of Chemical Physiology, The Scripps Research Institute, La Jolla, California, United States of America; University of California San Francisco, United States of America

## Abstract

Heterochromatin preferentially assembles at repetitive DNA elements, playing roles in transcriptional silencing, recombination suppression, and chromosome segregation. The RNAi machinery is required for heterochromatin assembly in a diverse range of organisms. In fission yeast, RNA splicing factors are also required for pericentric heterochromatin assembly, and a prevailing model is that splicing factors provide a platform for siRNA generation independently of their splicing activity. Here, by screening the fission yeast deletion library, we discovered four novel splicing factors that are required for pericentric heterochromatin assembly. Sequencing total cellular RNAs from the strongest of these mutants, *cwf14Δ*, showed intron retention in mRNAs of several RNAi factors. Moreover, introducing cDNA versions of RNAi factors significantly restored pericentric heterochromatin in splicing mutants. We also found that mutations of splicing factors resulted in defective telomeric heterochromatin assembly and mis-splicing the mRNA of shelterin component Tpz1, and that replacement of *tpz1^+^* with its cDNA partially rescued heterochromatin defects at telomeres in splicing mutants. Thus, proper splicing of RNAi and shelterin factors contributes to heterochromatin assembly at pericentric regions and telomeres.

## Author Summary

Heterochromatin formation at specific genomic regions is critical for processes as diverse as gene expression and chromosome segregation. The formation of silent heterochromatin at repetitive DNA elements requires processing of transcripts by the RNA interference machinery. Curiously, factors involved in proper RNA splicing are required for heterochromatin assembly, and it was proposed that splicing factors provide a platform for the recruitment of RNAi complexes independently of their role in regulating splicing. In this study, we found several novel splicing factors involved in heterochromatin assembly. Our analysis of genome-wide splicing patterns by RNA sequencing showed that the mRNAs of RNAi factors are very sensitive to perturbations of RNA splicing machinery. Moreover, we found that splicing factors are critical for the production of a telomere shelterin component and proper telomeric heterochromatin assembly. Most importantly, we showed that introducing the cDNA versions of RNAi and shelterin components alleviates heterochromatin defects associated with splicing factor mutations. Thus splicing factors are required for heterochromatin assembly mainly by regulating the proper splicing of heterochromatin assembly factors.

## Introduction

Eukaryotic genomic DNA associates with histone and non-histone proteins to form chromatin, which is necessary for the spatial and temporal organization of chromosomes. A distinction is commonly drawn between two types of chromatin: euchromatin and heterochromatin. Euchromatin is less condensed and often associated with genes that are actively transcribed. Heterochromatin is highly condensed and often forms over repetitive DNA elements such as transposons. The formation of heterochromatin prevents expression of transposons, improper recombination of repetitive genomic loci, and missegregation of chromosomes during mitosis and meiosis, thus maintaining genome stability [1], [2].

Histones within heterochromatin regions are usually hypo-acetylated and methylated at histone H3 lysine 9, which serves as a binding site for heterochromatin protein 1 (HP1) [3]–[5]. HP1 subsequently recruits diverse proteins to regulate cellular processes such as transcriptional silencing, recombination suppression, and chromosome segregation [1]. The mechanism that attracts histone methyltransferases and deacetylases to repetitive DNA elements is under intensive study. In certain cases, sequence-specific DNA binding proteins are directly involved in recruitment of these enzymes [6]–[9]. Alternatively, the repetitive nature may itself be sufficient to trigger heterochromatin assembly [10], [11]. Recent work has shown that DNA repeats are transiently transcribed, and the transcripts are processed by the RNA interference (RNAi) machinery into small interfering RNAs (siRNAs), which help target histone-modifying enzymes to repeat regions. However, the mechanistic details of RNAi-mediated heterochromatin assembly is not yet well understood [12]–[14].

The mechanisms by which heterochromatin is assembled and regulated have been extensively studied in the fission yeast *S. pombe* because it shares basic pathways of heterochromatin assembly with mammals, yet has the key advantages of facile genetics and single representative genes for most key families of mammalian chromatin-modifying factors. In this organism, heterochromatin is present mainly at pericentric regions, subtelomeres, and the silent mating-type region, all of which contain similar repeat sequences composed of *dg* and *dh* repeats [1]. These repeats are transcribed during S-phase of the cell cycle by RNA polymerase II. The transcripts are sliced by Ago1 and then reverse-transcribed by the RNA-directed RNA polymerase complex (RDRC: Rdp1, Cid12, and Hrr1) into double stranded RNAs, which are processed by Dcr1 into small interfering RNAs (siRNAs). These siRNAs are loaded onto Argonaute siRNA chaperone complex (ARC: Arb1, Arb2, and Ago1) and then onto the RNA-induced transcriptional silencing complex (RITS: Ago1, Chp1, and Tas3). RITS is targeted to the repeat regions through base pairing between siRNAs and the nascent transcripts and recruits the H3K9 methyltransferase complex CLRC, which contains SET domain protein Clr4 as its catalytic subunit. H3K9 methylation recruits HP1 family proteins Swi6 and Chp2, which in turn recruit histone deacetylases (HDACs) such as SHREC to further compact chromatin (see review [12]–[14]).

Surprisingly, in addition to these complexes, splicing factors are required for heterochromatin assembly at pericentric regions [15]–[17]. In fission yeast, 43% of genes contain introns [18], indicating the prevalence of splicing in this organism. The spliceosome and the splicing reactions of fission yeast are also highly conserved with those of higher eukaryotes, which utilize snRNAs U1, U2, U4, U5, and U6, as well as over one hundred protein components (see review [19]). The process of splicing starts when U1 and U2 snRNPs bind to the 5′ splice site and branch point on a pre-mRNA, respectively. The U4/U6.U5 snRNP and the Prp19 complex (also known as NineTeen Complex, or NTC) are then recruited to form the precatalytic spliceosome. After the release of U1 and U4, the spliceosome is activated and the 5′ splice site is cleaved and fused to the branch point to form a lariat structure. Next, 3′ intron cleavage is coupled to exon ligation in a post-spliceosomal complex of U2, U5, and U6. The mature mRNA is then released and the snRNPs recycled. Most notably, temperature-sensitive mutants *prp10-1* (component of U2) and *cwf10-1* (component of U5) exhibit silencing defects at pericentric regions [15]. Like *dcr1Δ*, these mutants lose most siRNAs derived from pericentric repeats [15]. Additionally, the spliceosome associates with RDRC component Cid12 [15], [20], indicating a possible direct role of splicing factors in connecting the nascent transcripts and the RNAi machinery during heterochromatin formation. It was hypothesized that splicing factors act in the RNAi pathway independently of RNA splicing because silencing defects are obvious when the splicing of a control *tbp1* mRNA is intact and introducing cDNAs of *ago1^+^* or *hrr1^+^* was unable to rescue pericentric heterochromatin silencing defects of *prp10-1* cells [15], [17]. However, the possibility that splicing factors regulate the proper processing of RNAi factors has not been rigorously tested. Notably, a number of recently identified factors involved in RNAi (*arb1^+^*, *arb2^+^*, *ers1^+^*, and *dsh1^+^*) contain introns and might require splicing factors for their proper expression [21]–[24].

In this study, we performed a screen of the *S. pombe* nonessential gene deletion strain library and discovered four new putative splicing factors involved in pericentric heterochromatin assembly. We demonstrated that the phenotype of the strongest of these, *cwf14Δ*, is similar to those of RNAi mutants in regulating pericentric heterochromatin assembly. RNA-seq analyses further found that *cwf14Δ* resulted in mis-splicing of a subgroup of genes, including a number of RNAi factors. Moreover, we showed that introducing the cDNAs of three RNAi factors, *ago1^+^*, *arb2^+^*, and *ers1^+^*, significantly alleviated silencing defects associated with *cwf14Δ*. Furthermore, we found that the mRNA of telomere shelterin protein Tpz1, which is involved in telomeric silencing, was also mis-spliced in splicing mutants and that introducing *tpz1^+^* cDNA partially rescues telomeric silencing defects of splicing factor mutants. Thus splicing factors are involved in heterochromatin assembly mainly through regulating the proper splicing of heterochromatin assembly factors.

## Results

### A high-throughput screen for mutants affecting pericentric heterochromatin

**Figure 1:**
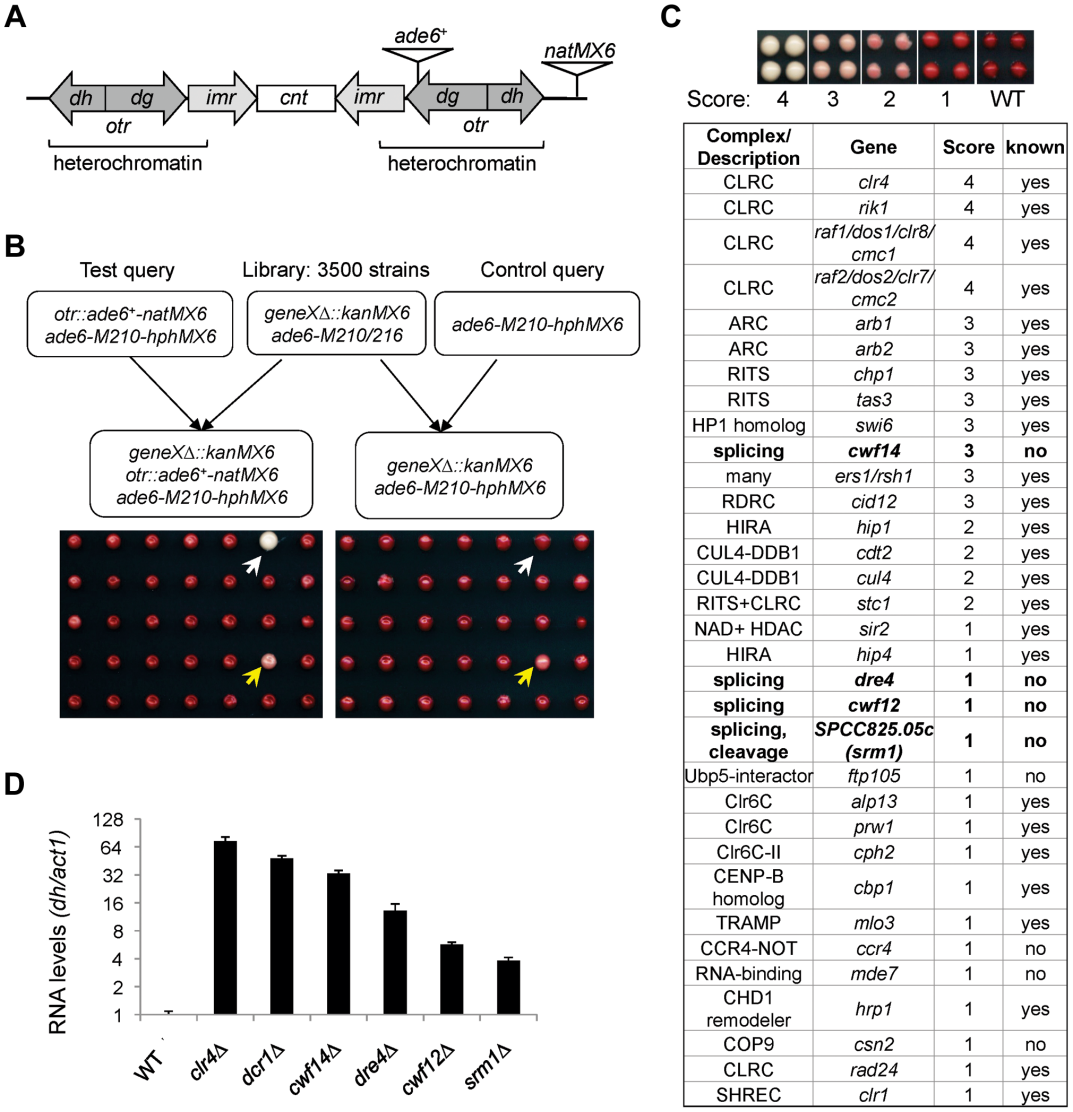
A genetic screen for nonessential genes required for pericentric heterochromatin silencing in fission yeast. (A) Schematic diagram of the *otr::ade6^+^-natMX6* reporter. (B) Workflow to introduce *otr::ade6^+^* into the deletion library. A control screen was performed in parallel. The selection of only *ade6-M210-hphMX6* progeny generates a uniform background for color development. White arrows denote a mutant that affects silencing, and yellows arrows denote a false positive that affects colony color independently of silencing. (C) List of mutants identified affecting pericentric silencing. Hit colonies were assigned color scores between 1 and 4, as indicated. (D) qRT-PCR analyses of transcripts derived from pericentric *dh* repeats, normalized to *act1*. Wild-type was set to 1. Error bars represent standard deviation of three experiments.

To comprehensively identify factors required for pericentric heterochromatin assembly, we performed a screen of the fission yeast haploid deletion library for mutants that affect silencing of a reporter inserted into pericentric heterochromatin, *otr::ade6^+^* (Figure 1A–C) [25]. In wild-type cells, *otr::ade6^+^* is silenced, causing red colony color on low adenine medium. However, when heterochromatic silencing is lost at the pericentric region, *otr::ade6^+^* is expressed, and colonies are white. Strains with intermediate silencing defects show variable degrees of pink/red color, allowing rough phenotypic quantification (Figure 1C). In order to eliminate strains that have inherent metabolic defects causing lighter-than-red color, we performed a control screen with an *ade6-M210* query strain lacking the *otr::ade6^+^* reporter (Figure 1B), which allowed us to filter false positives out of the screen. Our finalized list of hits is shown in Figure 1C. Each hit colony was assigned a score between 1 and 4, with 4 indicating the strongest silencing defects.

Among the mutants identified, 25 were previously known to be required for pericentric heterochromatin assembly, validating the effectiveness of our screen. These were mutants in the complexes of CLRC, ARC, RITS, RDRC, CUL4-DDB1, HIRA, Clr6C, TRAMP, and SHREC, and in individual factors such as HP1 homolog Swi6, NAD^+^ histone deacetylase Sir2, CENP-B homolog Cbp1, and CHD1 remodeler Hrp1 (Figure 1C). There were also a number of previously reported heterochromatin mutants that are listed in the library but were missed in our screen, such as *ago1Δ*, *rdp1Δ*, and *dcr1Δ*. We confirmed by PCR that the null mutation was missing in these strains, indicating that false negatives are more likely the result of incorrect deletions present in the library than due to methodological bias.

Most interestingly, there were eight novel mutants identified in this screen, of which four are uncharacterized genes implicated in various steps of mRNA splicing: *cwf14* (*SPBC24C6.11*), *dre4* (*SPAC13C5.02*), *cwf12* (*SPBC32F12.05c*), and the human SRRM1 homolog we named *srm1* (*SPCC825.05c*). Each of these strains showed elevated levels of pericentric *dh* transcripts, a common phenotype of heterochromatin mutants, suggesting that these mutants indeed affected pericentric heterochromatin assembly (Figure 1D). As *cwf14Δ* showed the strongest phenotype among these four splicing mutants, we chose it as the focus of our subsequent experiments.

### Cwf14 is required for RNAi-mediated pericentric heterochromatin assembly

**Figure 2:**
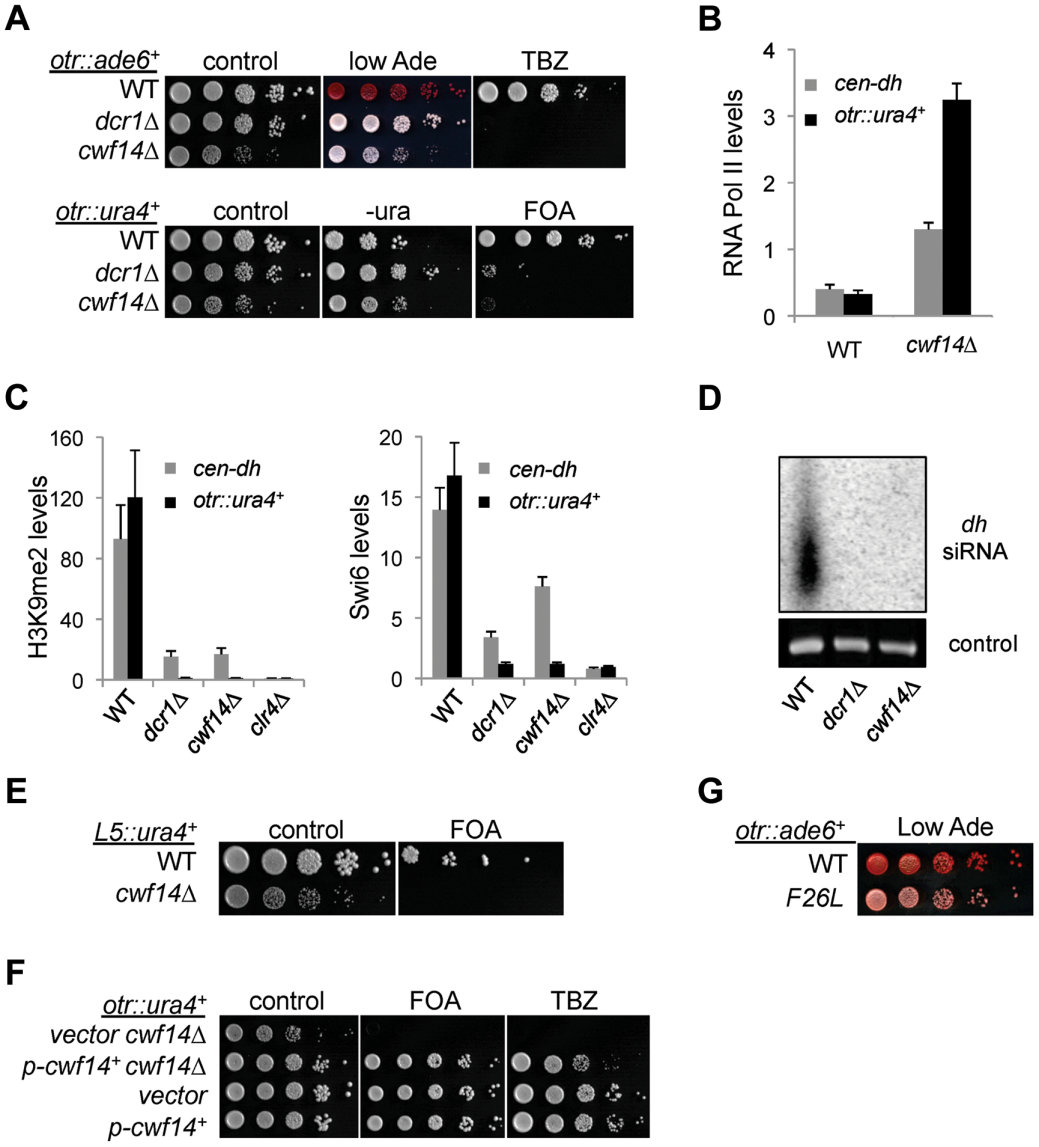
Cwf14 is required for pericentric heterochromatin assembly through the RNAi pathway. (A, E, F, and G) Serial dilution analyses to measure the expression of reporter genes and sensitivity to TBZ. (B and C) ChIP analyses of Pol II (Rpb1), H3K9me2, and Swi6 levels at pericentric *dh* repeats and *otr::ura4^+^*. Pol II ChIP was normalized to *act1* gene, and H3K9me2 and Swi6 ChIP was normalized to *act1* promoter. Error bars represent standard deviation of three experiments. (D) Northern blot analyses of siRNAs derived from pericentric *dh* repeats.

We first confirmed by serial dilution analysis that *cwf14Δ* cells containing *otr::ade6^+^* formed white colonies similar to those of *dcr1Δ* (Figure 2A). We also examined the effect of *cwf14Δ* on silencing of another reporter inserted at the same location, *otr::ura4^+^* [26]. The silencing of this reporter gene in wild-type cells allows them to grow on counterselective medium containing 5-fluoroorotic acid (FOA). Serial dilution analyses showed that *cwf14Δ* has comparable silencing defects to *dcr1Δ*, as indicated by attenuated growth on FOA medium (Figure 2A). Moreover, chromatin immunoprecipitation (ChIP) analyses showed increased enrichment of RNA Polymerase II at pericentric regions in *cwf14Δ* cells, indicating that the effect on pericentric transcript levels was at least in part due to increased transcription (Figure 2B).

Further ChIP analyses showed strong reduction in levels of heterochromatin hallmarks such as H3K9me2 and Swi6 at *otr::ura4^+^* and to a lesser extent at the endogenous *dh* repeats in *cwf14Δ* cells (Figure 2C). This pattern is similar to RNAi mutants such as *dcr1Δ*, but less pronounced than that of *clr4Δ*, suggesting that Cwf14 might regulate RNAi-mediated heterochromatin assembly. Consistent with this idea, siRNAs derived from pericentric repeats were eliminated in *cwf14Δ* cells, similar to *dcr1Δ* cells (Figure 2D).

It was shown that a 1.6 kb fragment of pericentric *dg* repeats (termed *L5*) induces heterochromatin assembly and silencing of adjacent genes when inserted into an ectopic site in an RNAi-dependent manner [27], [28]. Silencing is lost in *cwf14Δ* cells as well, consistent with the idea that Cwf14 is involved in RNAi-mediated heterochromatin assembly (Figure 2E).

Since pericentric heterochromatin promotes proper loading of cohesin near centromeres to promote chromosome bi-orientation [29]–[31], most heterochromatin mutants show defects in chromosome segregation [26] and sensitivity to the microtubule-destabilizing drug thiabendazole (TBZ) [32]. As expected, *cwf14Δ* was also sensitive to TBZ (Figure 2A). Moreover, both the silencing and TBZ-sensitivity phenotypes were rescued by complementation of *cwf14Δ* with a plasmid containing an intact copy of *cwf14* ^+^ under the control of its endogenous regulatory elements (Figure 2F). Since *cwf14Δ* cells had some growth defects, we also performed PCR-mediated random mutagenesis and isolated a mutant (*cwf14-F26L*) that resulted in silencing defects without significantly affecting growth (Figure 2G).

**Figure 3:**
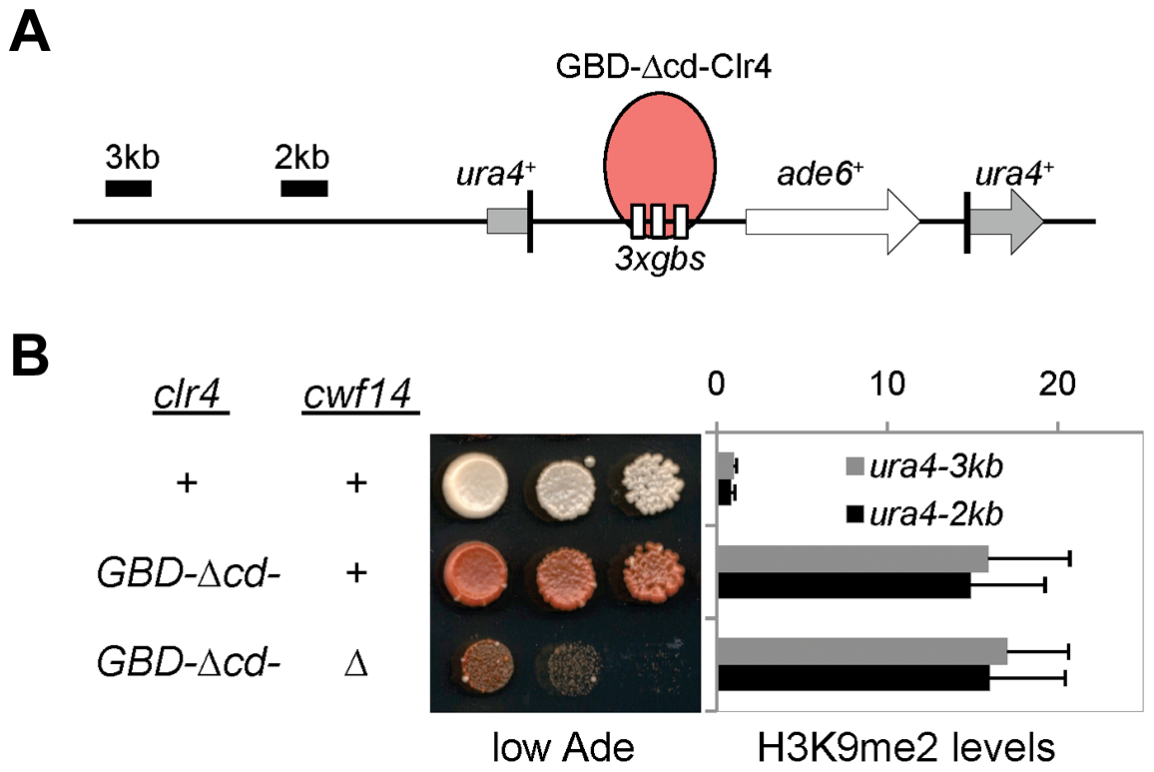
Cwf14 is not required for CLRC activity. (A) Schematic diagram of the *ura4::3xgbs-ade6^+^* reporter. (B) Left, serial dilution analysis to measure reporter gene expression. Right, ChIP analysis of H3K9me2 levels at the reporter, normalized to *act1* promoter. Error bars represent standard deviation of three experiments.

To test whether *cwf14Δ* affects H3K9 methyltransferase activity of CLRC, we used a system in which Clr4 is tethered via a fused Gal4 DNA binding domain (GBD) to an ectopically integrated *ade6^+^* reporter adjacent to three copies of the Gal4 binding site (*3xgbs-ade6^+^*) [33] (Figure 3A). This tethering induces heterochromatin formation over a 6 kb locus, silencing transcription of the *ade6^+^* reporter. RNAi factors are not required for this silencing, consistent with the idea that RNAi is required for the targeting of CLRC to DNA repeats [33]. Dilution analysis showed that *cwf14Δ* had no effect on Clr4-tethered silencing of *3xgbs*-*ade6^+^*, and ChIP analyses showed that H3K9me2 was enriched at nearby loci 2 and 3 kilobases away in *cwf14Δ* cells, similar to wild-type cells (Figure 3B). Collectively, these data suggest that Cwf14 is involved in heterochromatin formation at pericentric repeats through the RNAi pathway.

### Cwf14 associates with the spliceosome

**Figure 4:**
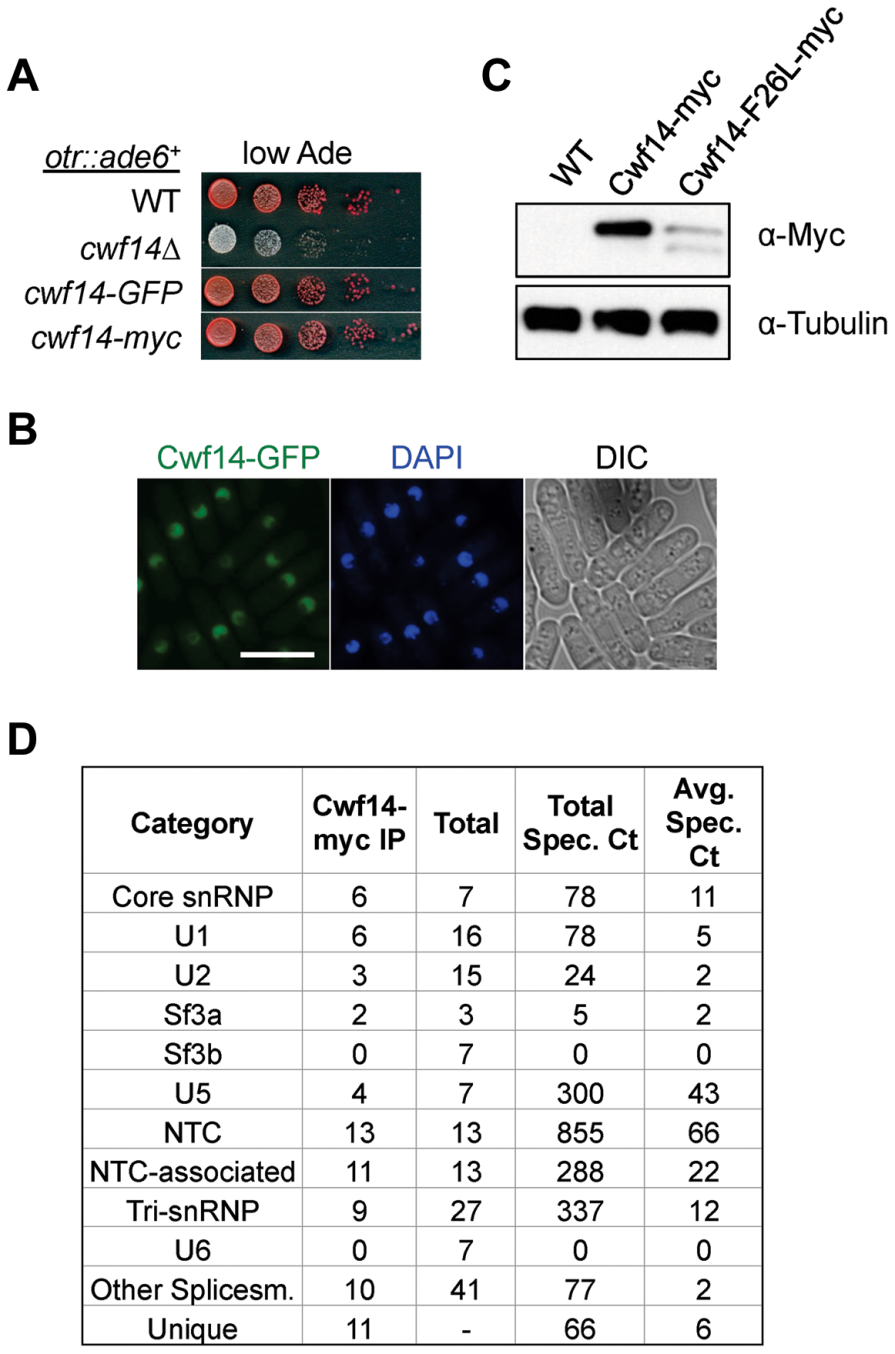
Cwf14 associates with the spliceosome. (A) Tagged versions of Cwf14 are functional as indicated by serial dilution analysis. (B) Imaging of cells expressing Cwf14-GFP, with DAPI to stain nucleus. Scale bar, 10 µm. (C) Western blot analysis of Cwf14-myc expression. (D) Mass spectrometry analyses of purified Cwf14-myc complex. Factors were binned into annotated subcomplexes.

Cwf14 is highly conserved across species. Its budding yeast homolog, Bud31p, co-purifies with the spliceosome [34], and *BUD31*Δ causes mis-splicing of the mRNAs of *ARP2* and *SRC1*, two factors required for proper budding [35]. Cwf14 was initially identified in a purification of splicing factor Cdc5, together with a number of spliceosome components [36]. However, whether Cwf14 is a stable component of the spliceosome and involved in splicing has not been tested directly. In order to further specify the mechanism of Cwf14 action, we constructed C-terminally tagged versions of *cwf14* at its endogenous locus. Cwf14-GFP and Cwf14-myc were fully functional, as they did not show silencing defects of *otr::ade6^+^* (Figure 4A). Imaging of Cwf14-GFP showed that Cwf14 localizes predominantly in the nucleus (Figure 4B). Western blot analysis showed that Cwf14-myc is a 40 kD protein. Moreover, the F26L mutation resulted in reduced Cwf14 protein levels, indicating that it is a partial loss-of-function allele (Figure 4C). We also performed immunoprecipitation of Cwf14-myc followed by MudPIT mass spectrometry to identify its interacting proteins. Most factors that co-immunoprecipitated with Cwf14, but not in a control purification, were components of the spliceosome, especially from subcomplexes NTC and U5 (Figure 4D and Table S1). This result corroborates data that Cwf14 co-precipitates with Prp17, Prp19, and Cwf2, all members of U5-associated NTC [19], [37], as well as a component of U5 snRNP, Cwf10 [38].

### Cwf14 is involved in splicing of specific mRNAs

Previous work suggests that the spliceosome is involved in heterochromatin assembly through tethering RDRC to pericentric transcripts [15]. This is because RDRC component Cid12 associates with the spliceosome [15], [20], and no splicing defects were observed in the well-characterized splicing substrate *tbp1* in splicing factor mutants when silencing defects were apparent [15], [17]. However, purifications of spliceosome components, including our purification of Cwf14, have not identified any other heterochromatin components [36], [37], [39] (Table S1). These results indicate either that the physical connection between the spliceosome and RDRC is very weak, or that Cid12 is present in two separate complexes, RDRC and spliceosome. It remains a possibility that splicing factors regulate the correct processing of mRNAs involved in RNAi-mediated heterochromatin assembly.

**Figure 5:**
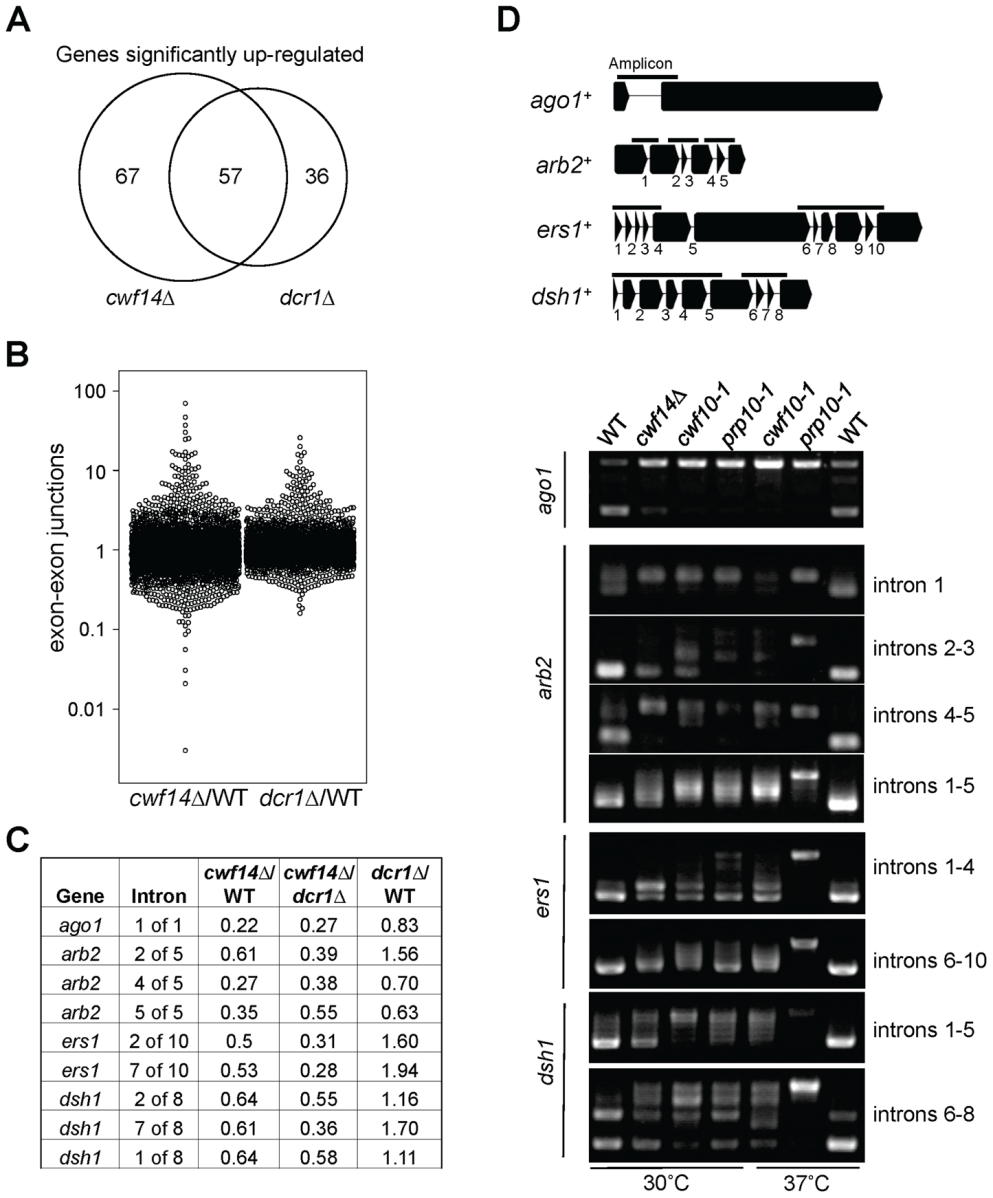
Cwf14 is required for the proper splicing of RNAi factors. (A) Area-proportional overlap of genes up-regulated in *cwf14Δ* and *dcr1Δ* (each compared to wild type) at p<0.05. (B) Ratios of exon-exon junction counts from RNA-seq for each junction in the indicated samples. A ratio of 1 indicates no difference. The bottom tail of *cwf14Δ*/WT, which is less pronounced in *dcr1Δ/*WT, indicates mis-splicing. (C) RNA-seq analysis shows mis-splicing of a number of RNAi factor introns in *cwf14Δ*. Ratios are exon-exon junction counts from RNA-seq for each junction in the indicated samples. (D) Top, diagram of RNAi genes with intron positions indicated. Bars represent PCR fragments used to analyze intron retention. Bottom, RT-PCR analyses of RNA with primers flanking introns.

In order to test whether *cwf14Δ* has a general splicing defect, we performed RNA-seq analyses of total cellular RNAs from wild-type, *dcr1Δ*, and *cwf14Δ* cells. The gene expression profiles of *cwf14Δ* and *dcr1Δ* showed strong overlap of significantly up-regulated genes (Figure 5A and Tables S2 and S3), consistent with the idea that Cwf14 functions in the RNAi pathway. We found that *cwf14Δ* indeed resulted in intron retention of a portion of genes (Figure 5B and Table S4), consistent with the finding that it is associated with the spliceosome. The majority of introns were properly processed, which indicates that Cwf14 only moderately affected the activity of the spliceosome. Interestingly, many RNAi factors contain introns. Although unbiased ranking of exon-exon junction ratios between wild-type and *cwf14Δ* RNAs did not show preferential enrichment of RNAi or heterochromatin assembly factors (Table S4), significant intron retention was observed within mRNAs from *ago1*, *arb2*, *ers1*, and *dsh1* in *cwf14Δ* cells as compared to those in wild-type cells, despite similar sequencing depths genome-wide and similar levels of each gene transcript in both samples (Figure 5C). RT-PCR analyses confirmed that these mRNAs were indeed spliced inefficiently in *cwf14Δ* as well as in *cwf10-1* and *prp10-1* cells (Figure 5D). Moreover, Western blot analysis showed strong reduction of protein levels of Flag-Ago1, the RNAi factor most severely affected by *cwf14Δ*, in both *cwf14Δ* and *cwf10-1* strains (Figure S1), indicating that the mis-splicing of *ago1* mRNA, and possibly other RNAi factors as well, resulted in altered protein levels.

**Figure 6:**
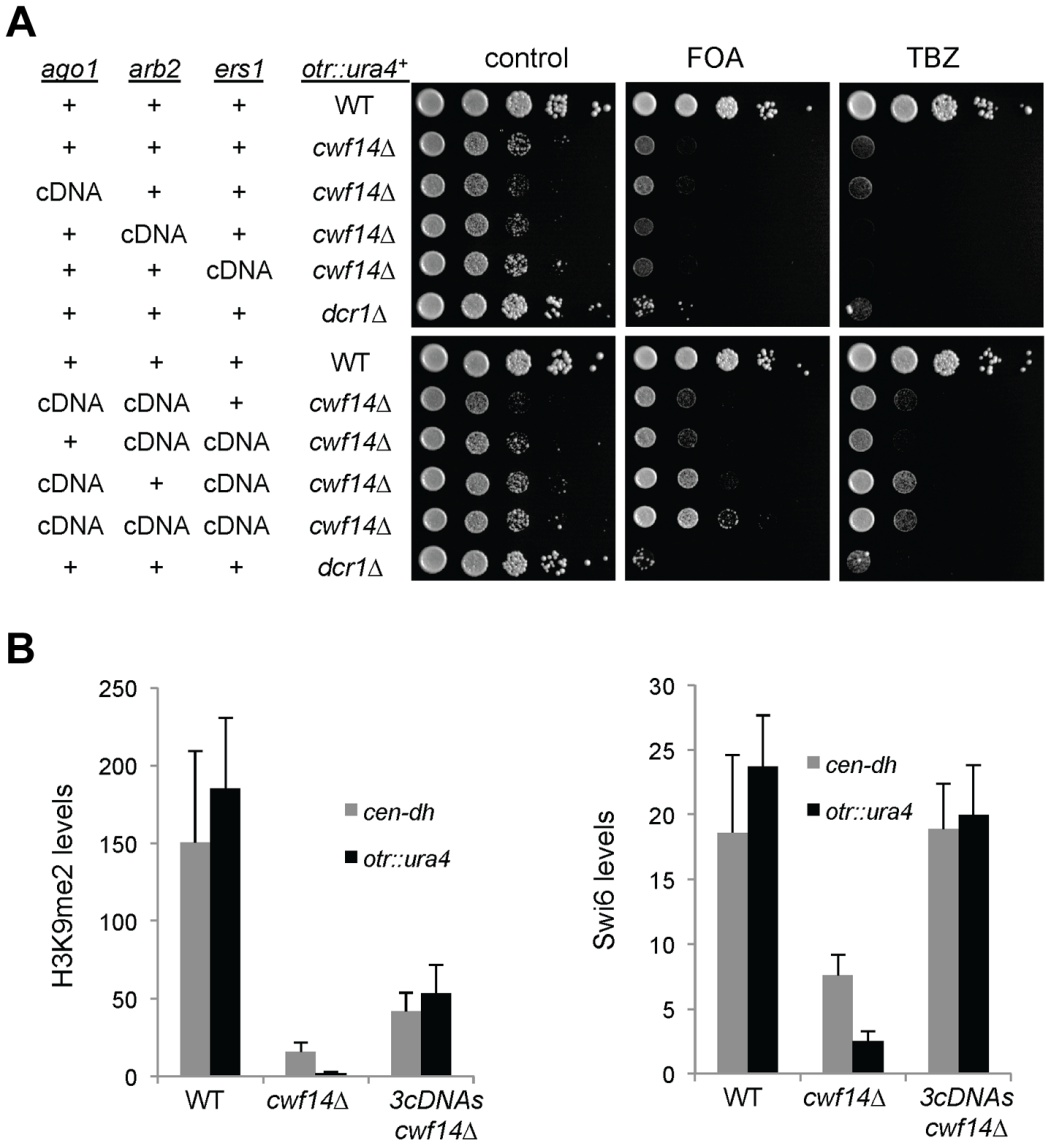
Introducing cDNAs of RNAi factors rescues pericentric heterochromatin defects of *cwf14Δ* cells. (A) Serial dilution analyses to measure the expression of *otr::ura4^+^* and sensitivity to TBZ. (B) ChIP analyses of H3K9me2 and Swi6 levels at pericentric *dh* repeats and *otr::ura4^+^*, normalized to an *act1* promoter fragment. Error bars represent standard deviation of three experiments.

Previously, it was shown that introducing cDNA versions of *ago1^+^* or *hrr1^+^* failed to rescue silencing defects of *prp10-1* [15]. We also generated a cDNA version of *ago1^+^* at its endogenous chromosomal location and under the control of its endogenous regulatory elements (*ago1^+^::cDNA*). This cDNA construct showed no defects in silencing of *otr::ura4^+^*, indicating that this replacement created a functional Ago1 protein (Figure S2). However, *ago1^+^::cDNA* was unable to rescue *otr::ura4^+^* silencing defects and TBZ sensitivity of *cwf14Δ* (Figure 6A), even though it restored Flag-Ago1 protein levels (Figure S1). We reasoned that the inability of *ago1^+^::cDNA* to rescue *cwf14Δ* defects is because other RNAi factors are also mis-spliced. We thus generated cDNA versions of *arb2^+^* and *ers1^+^* at their endogenous chromosomal loci, which were both functional (Figure S2). Neither *arb2^+^::cDNA* nor *ers1^+^::cDNA* alone showed any effect on silencing of *otr::ura4^+^* in *cwf14Δ* cells (Figure 6A). However, when combinations of two cDNAs were introduced together into *cwf14Δ* cells, there was a detectable rescue of silencing defects and TBZ sensitivity, and the effect was stronger when all three cDNAs were introduced (Figure 6A). Further ChIP analysis showed that both H3K9me and Swi6 protein levels were significantly increased at both *otr::ura4^+^* and pericentric *dh* repeats in *cwf14Δ* cells supplemented with *ago1^+^*, *arb2^+^*, and *ers1^+^* cDNAs (3cDNAs) (Figure 6B).

We also found that introducing *ago1^+^::cDNA* was sufficient to rescue the silencing defects of *otr::ade6^+^* associated with *cwf10-1* (Figure S3A). Moreover, there is a significant increase in both H3K9me and Swi6 levels at both *otr::ade6^+^* and *dh* repeats in *cwf10-1 ago1^+^::cDNA* cells (Figure S3B). Altogether, these results suggest that mutations in different splicing factors affect the splicing of diverse RNAi factors to regulate heterochromatin assembly at pericentric regions.

Thus our results clearly demonstrated that splicing factors mainly exert their effects on pericentric heterochromatin assembly by promoting the proper splicing of RNAi factors. However, we caution that the rescue of heterochromatin silencing defects of *cwf14Δ* cells was incomplete even with *ago1^+^*, *arb2^+^*, and *ers1^+^* cDNAs. This probably reflects the requirement of Cwf14 for the proper splicing of other RNAi factors such as *dsh1^+^*, *rdp1^+^*, *arb1^+^*, *hrr1^+^*, or some unidentified factors involved in heterochromatin assembly. Such an idea is supported by the fact that the rescue of *cwf14Δ* silencing defects correlated with the number of cDNA constructs that were introduced. It is also possible that splicing factors have a direct contribution in pericentric heterochromatin assembly. However, the strong rescue of pericentric silencing in *cwf14Δ* cells with cDNA constructs suggests that the regulation of RNAi factor splicing is a major role of splicing factors in this process.

### Cwf14 regulates the proper splicing of *tpz1* to control telomere silencing

**Figure 7:**
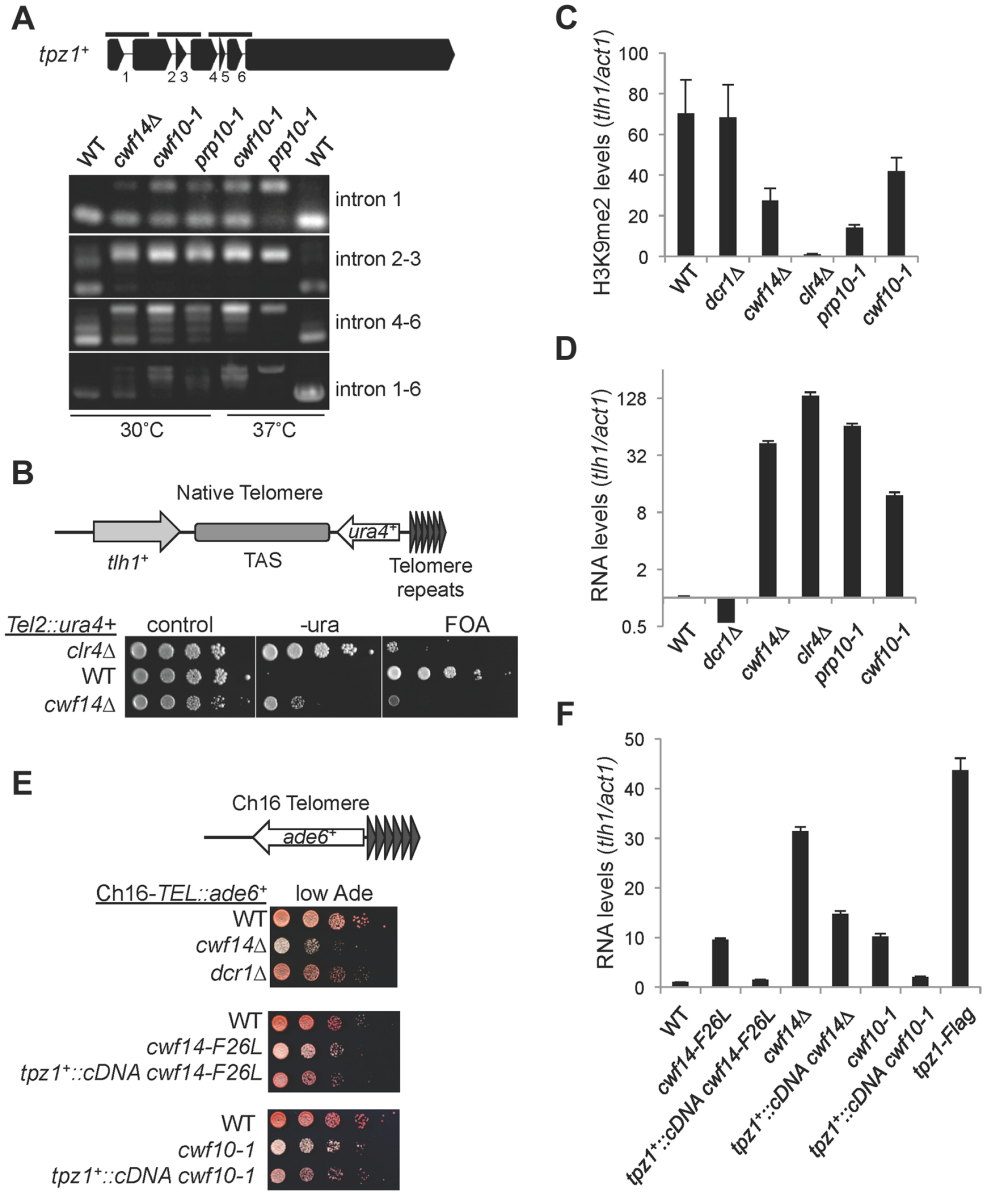
Splicing factors regulate the proper splicing of shelterin component Tpz1. (A) RT-PCR analyses of RNA with primers flanking introns. (B, E) Serial dilution analyses to measure reporter gene expression. (C) ChIP analyses of H3K9me2 levels, normalized to *act1* promoter. Error bars represent standard deviation of three experiments. (D, F) qRT-PCR analyses of transcripts derived from *tlh1*, normalized to *act1* gene. Wild type was set to 1. Error bars represent standard deviation of three experiments.

We also found that telomere shelterin component *tpz1^+^* showed a strong reduction in exon-exon junction sequencing reads in *cwf14Δ* cells relative to wild-type cells, and this phenotype was confirmed by RT-PCR analyses in *cwf10-1* and *prp10-1* as well (Figure 7A). A C-terminally Flag-tagged version of Tpz1 [40] affected silencing of a reporter gene inserted near telomere repeats (Figure S4), suggesting that Tpz1 is required for telomere silencing. We then analyzed the effect of splicing factor mutations on silencing of a reporter inserted near telomere repeats of chromosome two (*Tel2::ura4^+^*) [41]. Indeed, *cwf14Δ* resulted in silencing defects at this reporter gene (Figure 7B), accompanied by a reduction of H3K9me levels at *tlh1*, which is embedded at telomeric heterochromatin, as well as the accumulation of *tlh1* transcripts (Figure 7C and 7D). Both *cwf10-1* and *prp10-1* cells showed increased *tlh1* transcript levels, indicating that loss of telomeric silencing is a general feature of splicing mutants (Figure 7C and 7D). Because multiple DNA sequences contribute to heterochromatin assembly at telomeres, including *tlh1^+^*, telomere associated sequences (TAS), and terminal *TEL* repeats [8], we further tested silencing at *TEL::ade6^+^*, which is inserted on the mini-chromosome Ch16 adjacent to telomere repeats [42]. We found that *cwf14Δ*, *cwf14-F26L*, and *cwf10-1* resulted in loss of silencing of this reporter (Figure 7E). Interestingly, replacement of *tpz1^+^* with its cDNA partially rescued telomeric silencing phenotypes of the *cwf14-F26L* and *cwf10-1* mutants (Figure 7E and 7F). We could only marginally rescue the telomere silencing defects of *cwf14Δ* cells with *tpz1^+^::cDNA* (Figure 7F and data not shown). Thus it is possible that additional factors involved in telomere silencing might also contain introns and depend on the spliceosome to properly regulate their splicing. Alternatively, splicing factors might affect telomere silencing through additional mechanisms. Nonetheless, these results demonstrate that inefficient splicing of *tpz1* contributes to telomeric silencing defects in splicing mutants.

## Discussion

The formation of heterochromatin requires RNAi-mediated processing of repeat-derived transcripts and the targeting of histone modifying activities to repeat regions, leading to H3K9me and the recruitment of HP1 proteins. It has been shown that splicing factors are required for RNAi-mediated heterochromatin assembly in fission yeast, although the mechanism by which they are involved is not well characterized. The previously prevailing model was that the spliceosome physically associates with RNAi factors to regulate heterochromatin assembly, rather than acting through its splicing activity [15], [16]. One of the main evidences for this idea is that splicing mutants show severe silencing defects even though the splicing of *tbp1* mRNA was not affected [15], [17]. However, whether these splicing factor mutants selectively affect the splicing of RNAi factors has not been rigorously tested.

Our RNA-seq analyses showed prominent intron retention of a subgroup of mRNAs in *cwf14Δ* cells, even though the majority of introns (including those of *tbp1*) are still properly processed (Figure 5B and Table S4). Interestingly, a number of key RNAi factors were among the list of strongly mis-spliced introns, a result that is further corroborated by RT-PCR analyses of a selective set of RNAi factor mRNAs in other spliceosome mutants such as *cwf10-1* and *prp10-1* (Figure 5D). Most importantly, we found that introducing a combination of cDNAs of RNAi factors significantly alleviated pericentric heterochromatin defects associated with *cwf14Δ* and *cwf10-1* (Figure 6 and S3). Thus splicing factors regulate the proper splicing of RNAi factors, which is a major, if not sole, contributor to heterochromatin assembly defects in splicing mutants. That introducing *tpz1^+^::cDNA* was able to partially rescue telomere silencing defects associated with splicing factors further supports the idea that mis-splicing of heterochromatin factors is the reason splicing factor mutants show heterochromatin assembly defects (Figure 7E and 7F).

It is noteworthy that *cwf14Δ* has only moderate splicing defects, with some introns show very strong sensitivity, whereas most others show little to no defects (Figure 5B and Table S4). This raises the question of whether specific intron sensitivity is a result of introns that are inherently difficult to splice. Consistent with this idea, our RT-PCR analyses showed prominent unspliced precursor mRNAs of RNAi factors even in wild-type cells (Figure 5D). It is also a striking pattern that heterochromatin factors in the same complexes tend to either have or not have introns. For example, many members of RNAi, such as *ago1^+^*, *arb1^+^*, *arb2^+^*, *ers1^+^*, *dsh1^+^*, *rdp1^+^*, and *hrr1^+^*, have introns, but none of CLRC (*clr4^+^*, *rik1^+^*, *raf1^+^*, *raf2^+^*, *cul4^+^*, *stc1^+^*), SHREC (*clr1^+^*, *clr2^+^*, *clr3^+^*, *mit1^+^*), or *swi6^+^* have any introns, raising the possibility of selective regulation of the RNAi pathway through general or specific changes in splicing efficiency. In fact, an analysis of splicing changes during meiosis showed that one intron of *arb1^+^* is induced to be spliced, while a different intron exhibits splicing repression [43]. Since splicing factors have been identified in screens that affect RNAi-based processes in worms, flies, and plants [44]–[48], it seems a conserved mechanism that proper splicing of the mRNAs of RNAi factors regulates the efficiency of RNAi inside the cell.

## Materials and Methods

### Genetic screens of the fission yeast deletion library

The Bioneer library is composed of strains of mixed endogenous *ade6* alleles: *ade6-M216*, which forms pink colonies on low adenine medium, and *ade6-M210*, which give rise to red colonies, similar to *ade6Δ*. To avoid the complication of *ade6-M216* alleles in our screen, we included in the query strain an *ade6-M210-mCherry* allele linked to a *hphMX6* cassette, which confers resistance to the antibiotic hygromycin B, allowing us to generate a uniform *ade6-M210* background. Screens were carried out according to previous protocols [49], with slight modifications. Yeast strains arrayed in 384 strains/plate format were pinned on YES agar medium containing 100 µg/ml G418, and *otr::ade6^+^-natMX6 ade6-210-hphMX6* and *ade6-210-hphMX6* strains were pinned on YES+100 µg/ml hygromycin B. After two days growth, strains were mated on SPA agar medium. Plates were incubated at 25°C for 3 days, then 42°C for 3 more days to kill vegetative cells. Strains were then germinated and correct genotype selected by pinning to YES+GNH (G418, Nat, and Hygromycin) or YES+GH (G418 and Hygromycin) medium, and then pinned to YE (low ade) medium for color readout.

### Fission yeast strains and genetic analyses

Cwf14-GFP and Cwf14-13myc strains were constructed by a PCR-based module method. *cwf14Δ*, *cwf12Δ*, *dre4Δ*, and *srm1Δ* were derived from the Bioneer fission yeast deletion library, verified via PCR, and backcrossed. The *p-cwf14^+^* plasmid was constructed by insertion of a PCR product containing *cwf14^+^* promoter and coding region into SphI and XmaI sites of pREP41. The *cwf14-F26L-myc* mutant strain was constructed by integrating a *cwf14^+^-myc-kanMX6* cassette amplified by error-prone PCR (high dNTP concentration) into the endogenous *cwf14^+^* locus. *ago1^+^::cDNA*, *arb2^+^::cDNA*, and *ers1^+^::cDNA* strains were constructed by replacing the endogenous genes with intronless cDNA versions. All were sequenced to confirm full replacement and lack of mutation. *cwf10-1* and *prp10-1* strains were a kind gift from Robin Allshire. Genetic crosses were used to construct all other strains. The genotype of strains is provided in Table S5. For serial dilution plating assays, ten-fold dilutions of mid-log-phase culture were plated on the indicated medium and grown for 3 days at 30°C unless otherwise indicated.

### RNA analyses

Total cellular RNA was isolated from log-phase cells using MasterPure yeast RNA purification kit (Epicentre) according to the manufacturer's protocol. Quantification with qRT-PCR was performed with Power SYBR Green RNA-to-CT one-step Kit (Applied Biosystems). RNA serial dilutions were used as templates to generate the standard curve of amplification for each pair of primers, and the relative concentration of target sequence was calculated accordingly. An *act1* fragment served as reference to normalize the concentration of samples. Sequence of DNA oligos is provided in Table S6. For RNA-seq, purified RNA was prepared by TruSeq Stranded Total RNA Kit (Illumina), which includes rRNA depletion and chemical fragmentation. Index adapters were added to allow for multiplexing. Paired-end sequencing with 100 bp read lengths was performed on Illumina HiSeq. Mapping was performed with the Tuxedo Suite consisting of Bowtie, TopHat, and Cufflinks. For *cwf14Δ*, 57,521,513 reads were obtained, and 83% mapped to the genome via Bowtie. For WT, 47,636,518 reads were obtained, and 82% mapped. For *dcr1Δ*, 64,972,007 reads were obtained, and 83% mapped. RNA-seq data have been deposited to the Sequence Read Archive (http://www.ncbi.nlm.nih.gov/sra/) with accession number SRP040479. For the dot plot, exon-exon junction ratios were filtered to remove several types: 1) junctions which mapped zero times in any sample (possible mapping noise), 2) junctions whose sum in WT, *cwf14Δ*, and *dcr1Δ* was less than 30 (to avoid randomness due to small sample sizes), and 3) ratios whose values were greater than 150 (to focus the diagram on splicing reduction). Northern blot of siRNAs was performed as described previously [50].

### Chromatin immunoprecipitation (ChIP) analysis

ChIP analyses were performed as described previously [51]. Antibodies used were H3K9me2 (Abcam 1220), Swi6 [52], and RNA Pol II (Covance 8WG16). Quantification with qPCR was performed with Maxima SYBR Green/ROX qPCR Master Mix (Thermo). Enrichment was calculated as: relative levels in ChIP/relative levels in total DNA. An *act1* promoter fragment was used as a control for normalization unless otherwise indicated. Sequence of DNA oligos was provided in Table S6.

### Protein purification and mass spectrometry analysis

Exponentially growing yeast cells were harvested, washed with 2xHC buffer (300 mM HEPES-KOH pH 7.6, 2 mM EDTA, 100 mM KCl, 20% glycerol, 0.1% NP-40, 2 mM DTT, and protease inhibitor cocktail (Roche)) and frozen in liquid nitrogen. Crude cell extracts were prepared by vigorously blending frozen yeast cells with dry ice using a household blender, followed by sonication and incubation with 30 ml 1xHC buffer containing 250 mM KCl for 30 min. The lysate was cleared by centrifugation at 82,700×*g* for 3 hours. The supernatants were incubated with 50 µl of C-myc antibody (Sigma C3956) overnight, for three hours the next day with 50 µl protein G agarose beads, washed eight times with 1xHC containing 250 mM KCl, then two times with the same buffer without NP-40. For mass spectrometry analysis, bound proteins were eluted with 2×100 µl of 50 mM Tris pH 7.5, 5% SDS, 5% glycerol, 50 mM DTT. MudPIT mass spectrometry analysis was performed as described previously [53].

## Supporting Information

Figure S1 Western blot analysis of Flag-Ago1 protein levels. Cell lysates were first immunoprecipitated with Flag-agarose beads and Western blot analyses were performed with a Flag antibody.
(PDF)

Figure S2 cDNA versions of RNAi factors are functional. Serial dilution analysis of cells to measure the expression of *otr::ura4^+^* and sensitivity to TBZ. Pictures for *dcr1Δ* are from the same plates as other strains.
(PDF)

Figure S3 Introducing *ago1^+^::cDNA* significantly rescues silencing defects associated with *cwf10-1*. (A) Serial dilution analysis of cells on low adenine medium to measure the expression of *otr::ade6^+^*. (B) ChIP analysis of H3K9 and Swi6 levels at *otr::ade6^+^*, normalized to an *act1* fragment. Error bars represent standard deviation of three experiments.
(PDF)

Figure S4 Tpz1 is required for telomere silencing. Serial dilution analysis of cells to measure the expression of *TEL::ura4^+^*, which is located near telomere repeats on Ch16.
(PDF)

Table S1 Mass spectrometry analysis of Cwf14-myc associated proteins.
(XLSX)

Table S2 Comparison of gene expression in wild type and *cwf14Δ* by RNA-seq.
(XLSX)

Table S3 Comparison of gene expression in wild type and *dcr1Δ* by RNA-seq.
(XLSX)

Table S4 Exon-exon junction counts and ratios across all samples.
(XLSX)

Table S5 Strains used in this study.
(XLSX)

Table S6 DNA oligos used in this study.
(XLSX)
