# Supplementary figures and images for: The Proper Splicing of RNAi Factors Is Critical for Pericentric Heterochromatin Assembly in Fission Yeast
Source: PLoS Genet. 2014 May 29;10(5):e1004334. doi: 10.1371/journal.pgen.1004334 (PMC4038458; doi:10.1371/journal.pgen.1004334)

Figure S1

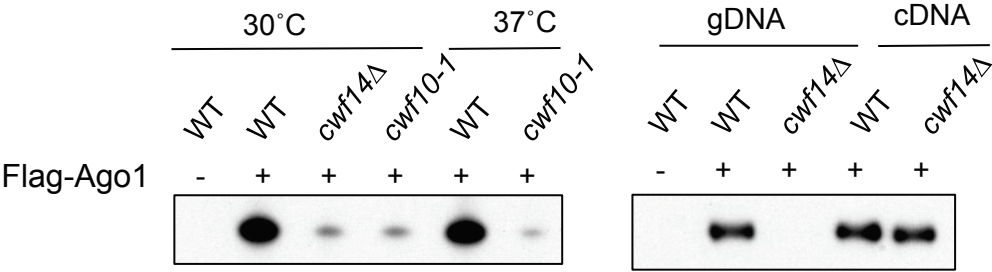

Supplement: Figure S1 — Western blot analysis of Flag-Ago1 protein levels. Cell lysates were first immunoprecipitated with Flag-agarose beads and Western blot analyses were performed with a Flag antibody. (PDF) [file pgen.1004334.s001.pdf]

Figure S2

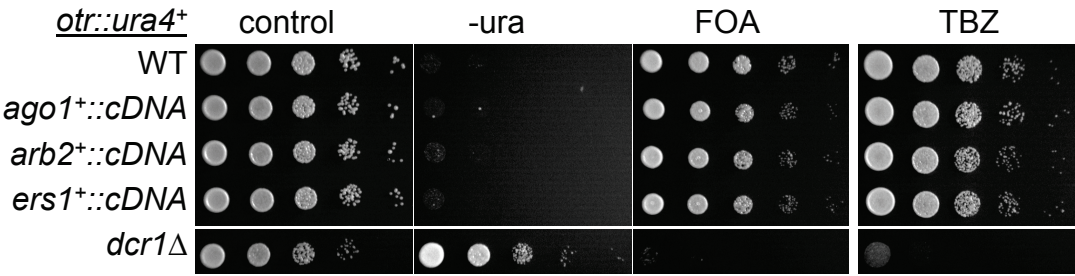

Supplement: Figure S2 — cDNA versions of RNAi factors are functional. Serial dilution analysis of cells to measure the expression of otr::ura4+ and sensitivity to TBZ. Pictures for dcr1Δ are from the same plates as other strains. (PDF) [file pgen.1004334.s002.pdf]

Figure S3

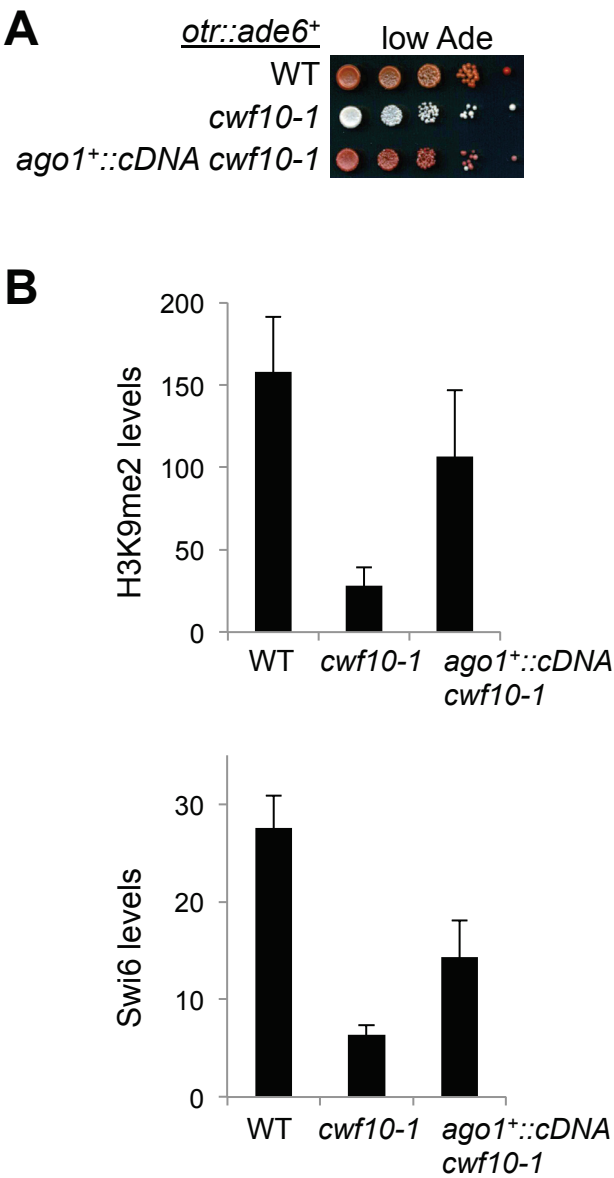

Supplement: Figure S3 — Introducing ago1+::cDNA significantly rescues silencing defects associated with cwf10-1. (A) Serial dilution analysis of cells on low adenine medium to measure the expression of otr::ade6+. (B) ChIP analysis of H3K9 and Swi6 levels at otr::ade6+, normalized to an act1 fragment. Error bars represent standard deviation of three experiments. (PDF) [file pgen.1004334.s003.pdf]

Figure S4

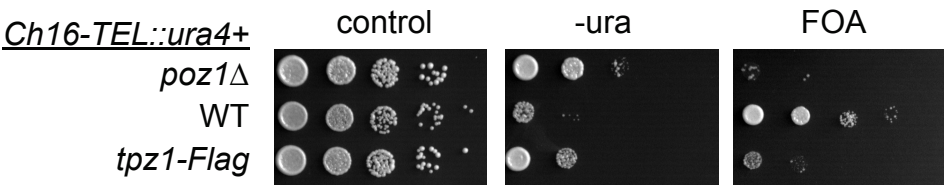

Supplement: Figure S4 — Tpz1 is required for telomere silencing. Serial dilution analysis of cells to measure the expression of TEL::ura4+, which is located near telomere repeats on Ch16. (PDF) [file pgen.1004334.s004.pdf]
